# Sex differences in primary hypertension

**DOI:** 10.1186/2042-6410-3-7

**Published:** 2012-03-14

**Authors:** Kathryn Sandberg, Hong Ji

**Affiliations:** 1 Center for the Study of Sex Differences in Health, Disease and Aging Georgetown University, Washington, DC 20057

## Abstract

Men have higher blood pressure than women through much of life regardless of race and ethnicity. This is a robust and highly conserved sex difference that it is also observed across species including dogs, rats, mice and chickens and it is found in induced, genetic and transgenic animal models of hypertension. Not only do the differences between the ovarian and testicular hormonal milieu contribute to this sexual dimorphism in blood pressure, the sex chromosomes also play a role in and of themselves. This review primarily focuses on epidemiological studies of blood pressure in men and women and experimental models of hypertension in both sexes. Gaps in current knowledge regarding what underlie male-female differences in blood pressure control are discussed. Elucidating the mechanisms underlying sex differences in hypertension may lead to the development of anti-hypertensives tailored to one's sex and ultimately to improved therapeutic strategies for treating this disease and preventing its devastating consequences.

## Introduction

Sex differences in biology arise from differences in sex chromosome dosage (2X vs. 1X or 0Y vs. 1Y) [1]. It is the transcription factor *Sry *located on the Y chromosome that causes differentiation of testes and leading to a rise in the levels of testosterone (T) *in utero*. Since one's sex is defined phenotypically (i.e., whether one is born with testes or ovaries), a functioning *Sry *leads to the birth of a male while in its absence (e.g., an XX or XO individual), ovaries develop and a female is born. Thus, Y dosage (0Y or 1Y) dictates the hormonal milieu *in utero *and throughout the life span. Physiological sex differences can also arise from differences in X dosage (2X or 1X) as a result of differences in parental imprinting, escape from X-inactivation and X-mosacism. This review will focus not only on the impact of the gonadal hormone milieu but also on the other effects that differences in the sex chromosome complement can exert on blood pressure control. The current state of knowledge regarding sex differences in the pathophysiology of human hypertension as well as the clinical implications of studies conducted in experimental animal models of hypertension will be addressed.

Many organizations including the leading United States authority on blood pressure - the Joint National Committee on the Prevention, Detection, Evaluation and Treatment of High Blood Pressure (JNC) - define hypertension as a systolic blood pressure (SBP) ≥ 140 or a diastolic blood pressure (DBP) of ≥ 90 mm Hg. According to the 7th and the latest report of the JNC [2], high blood pressure is divided into three categories (in mm Hg): Prehypertension (SBP of 120-139 or DBP between 80-89); Stage 1 hypertension (SBP of 140-159 or DBP between 90-99); and, Stage 2 hypertension (SBP ≥ 160 or DBP ≥ 100).

High blood pressure can lead to substantial morbidity by damaging the function of critical organs including the brain, heart, blood vessels and kidney [3]. Hypertension is a leading cause of stroke [4,5] and can cause hypertensive encephalopathy [6,7] resulting in headaches, confusion and even convulsions. Sustained hypertension can lead to hypertensive retinopathy of the eye, which untreated can lead to blindness [8]. Hypertension can cause myocardial infarction [9] and hypertensive cardiomyopathy [10]. Chronic kidney failure is often a result of hypertension, as is hypertensive nephropathy [11,12]. Elevated blood pressure is associated with high blood sugar. Thus hypertension could also contribute to type II diabetes [13].

Hypertension is a global health concern. One billion people worldwide have hypertension [14] and sixty five million inhabitants in the United States alone require treatment for their hypertension [15]. In fact, suboptimal blood pressure (SBP > 120 mm Hg) is the number one attributable risk factor for death throughout the world and it is responsible for more than 60% of cardiovascular disease and 50% of ischemic heart disease [16,17]. What is particularly problematic is that the majority of hypertensive individuals do not have their blood pressure under control (< 140/90 mm Hg) including nearly half of the hypertensive population in the United States [2]. A greater understanding of sex differences in the physiology and pathophysiology of blood pressure control could yield treatments that are better tailored to the individual because one's sex is taken into account. Furthermore, elucidating the mechanisms responsible for gonadal and sex chromosome effects on blood pressure could lead to the development of novel and improved antihypertensive therapeutics for treating this debilitating disease.

Hypertension can be induced by secondary causes such as chronic renal disease, pheochromocytomas or sleep apnea; however, the vast majority (> 95%) of human hypertension is due to unknown causes and is referred to as "essential" or primary hypertension [2]. Blood pressure is salt-sensitive in the majority of individuals, i.e., modulated by changes in dietary sodium intake. Salt-sensitivity is markedly more prevalent in hypertensive African Americans at 72%, compared with a 56% prevalence in hypertensive whites [18]. This review will focus on sex differences in essential hypertension including salt-sensitive hypertension and not on the less frequent causes of hypertension.

### Sex differences in blood pressure across the human life span

A wealth of blood pressure data in men and women has been collected over the years. In 1947, Boynton and Todd [19] measured blood pressure in 75,258 university students, which revealed that both SBP and DBP were significantly higher in young men than in young women [SBP/DBP (mm Hg): men, 122/74.5 vs. women, 111/69.7]. The National Health and Nutrition Examination Survey (NHANES), which was established in the early 1960s by the National Center for Health Statistics, a part of the Centers for Disease Control (CDC), conducts nationally representative surveys via interviews and physical examinations of the United States population. Data gathered from April 1971 to June 1974 through the NHANES I study of 28,043 individuals aged 1-74 years old revealed that sex differences in blood pressure originates in adolescence starting between the ages of 12-17, at which time, the mean SBP and DBP for boys consistently exceeded that for girls [20]. In younger children (6-11 years old), no sex differences in SBP or DBP were detected.

**Figure 1:**
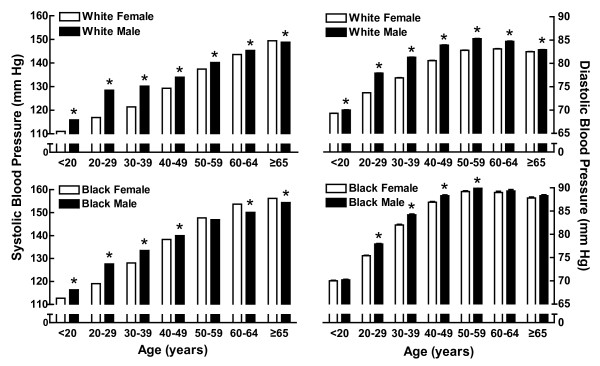
**Blood pressure in men and women across the life span**. Shown is SBP (left panels) and DBP (right panels) in white (upper panels) and black (lower panels) men (black bar) and women (white bar) from the Community Hypertension Evaluation Clinic program, which measured blood pressure in over 1 million people in the United States between 1973 to 1975. *p < 0.01 vs male (t-test). This figure was drawn using data from reference [21].

Between 1973-1975, one million Americans were screened in the Community Hypertension Evaluation Clinic Program [21] (Figure 1). Analysis of these data revealed that DBP was higher in men than in women in both Blacks and Whites across all age groups. White men had higher SBP than White women through their mid-sixties; however, after 65 years of age, the sex difference in SBP disappeared. Black men had higher SBP than Black women through the fifth decade of life but in older age groups, Black women had higher SBP than Black men. These racial differences in SBP across the life span may be related to differences in life expectancy. The CDC reported in October 2010 that life expectancy for non-Hispanic Black men (69.2 years) is 6.4 years shorter than non-Hispanic White men (75.6 years) [22]. Other factors could also contribute to these racial differences in blood pressure such as the rising epidemic of obesity. In this regard, it is of note that NHANES III (1988-1994) found that obesity and overweight prevalence were highest in non-Hispanic black women with more than half of this population of women aged 40 years or older being classified as obese and more than 80% as overweight [23].

**Figure 2:**
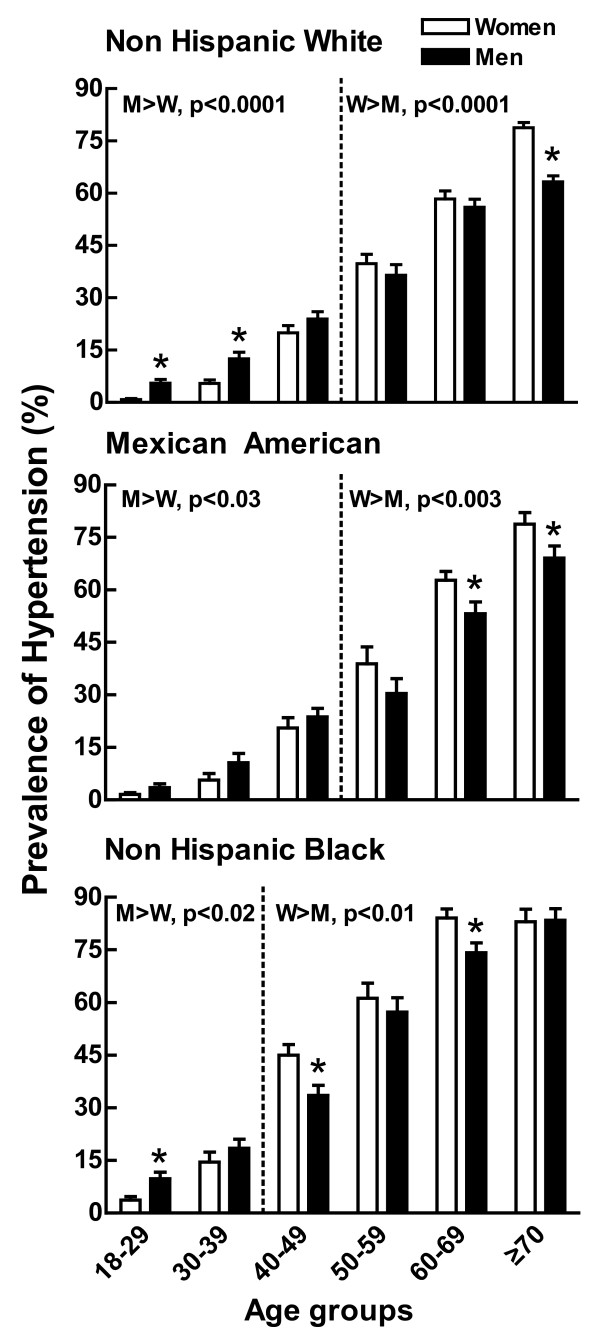
**Prevalence of hypertension in men and women as a function of age**. Shown is the prevalence of hypertension in non-Hispanic white (top panel), Mexican American (middle panel) and non-Hispanic black (bottom panel) man (black bar) and women (white bar) from 18 to > 70 years of age from data collected from the NHANES III examination between 1988-1991. This figure was drawn using data from reference [27].

Findings regarding sex differences in the prevalence of hypertension parallel the observations of sex differences in arterial blood pressure. NHANES, which surveyed data for the overall US population (excluding pregnant women, institutionalized individuals and military personnel) in all age and racial groups between 1999 and 2004 found that men regardless of race and ethnicity had a higher prevalence of hypertension than women in the 18-39 age group [24] (Figure 2). Between the ages of 50-69, the prevalence of hypertension remained greater in men than women in non-Hispanic whites while this sex difference was greatly diminished in the non-Hispanic Black population and disappeared in the Mexican Americans. After the age of 70, women had a higher prevalence of hypertension across all racial groups.

Several cross-sectional studies including the above Community Hypertension Evaluation Clinic Program [21], the Hypertension Detection and Follow-up Program Cooperative Group [25] and the NHANES II [26] and NHANES III [27] suggest that the rate of the age-associated increase in blood pressure accelerates in women around the fifth and sixth decades of life and eventually exceeds that of men (Figure 2). Whether or not blood pressure in women actually surpasses men at later ages remains controversial. Blood pressure in women did not exceed that in men at any age in the 30 year longitudinal study of 5,209 healthy men and women living in Framingham, Massachusetts between 30 and 60 years of age [28], nor was blood pressure higher in women in a 30 year longitudinal study of 202 men and women from a Pittsburgh high school population [29]. Longitudinal studies enable comparing the same study population over time and thus, age-associated traits are readily observed. In contrast, while cross-sectional studies have the advantage of acquiring data on huge populations (e.g., one million Americans), the population represented in their seventies likely has different characteristics than the population represented in their forties. For instance, it is known from the Framingham study that sex differences exist in the number of deaths due to hypertension-associated cardiovascular disease; coronary events occur twice as often in men than in women [30]. Thus, comparing the prevalence of hypertension in men and women across the life span would be confounded by the changes in representation of the surviving population.

In general, epidemiological studies indicate that the prevalence of hypertension is greater in men than women regardless of race, ethnicity or country of origin. The Hypertension Detection and Follow-up Program Cooperative Group screened 158,906 persons aged 30-69 years in 14 communities between 1973 to 1974 and found that hypertension was more prevalent in men than women of either European-American or African-American ancestry [31]. A secondary analysis of the prevalence of hypertension conducted on 46 population-based studies from 22 different countries during the period of 1960-1991 found the prevalence of hypertension in most but not all studies was higher in men than women. Age may well have been a factor in the studies that did not observe a lower prevalence rate of hypertension in women than men since age was not taken into account in many of these studies and varied from all ages with some ranging from 18-79 and 20-49 and 40-79 in others [32].

While mean sex differences in arterial pressure are not huge (e.g., sex differences in SBP rarely exceed 10 mm Hg and sex differences in DBP rarely exceed 5 mm Hg), it is important to note that an increase of 10 mm Hg SBP doubles the risk of developing cardiovascular disease while a drop of 5 mm Hg in SBP results in a 14% reduction in mortality due to stroke and a 9% reduction in mortality due to coronary heart disease [2]. Therefore, the observation that men have higher blood pressure than women, at least up to the fifth decade of life, has significant implications for pathophysiology. Moreover, deciphering the molecular mechanisms underlying sex differences in hypertension could ultimately lead to the discovery of new and improved antihypertensive treatments for both men and women.

### Sex differences in blood pressure in animals

**Figure 3:**
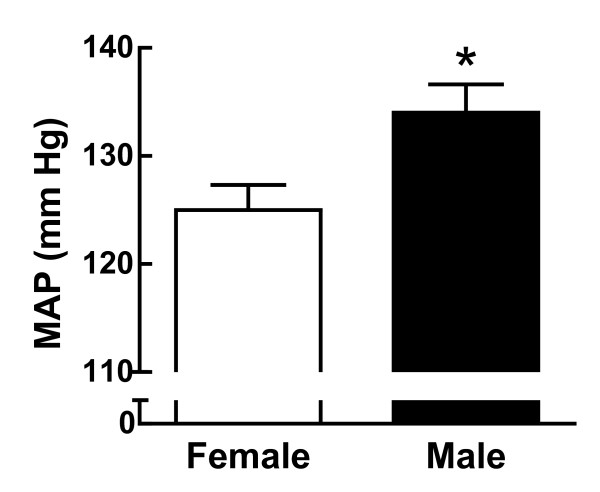
**Mean arterial pressure in female and male dogs**. Shown is the MAP in female (n = 80) and male (n = 67) street dogs of unknown age. *p < 0.05 vs female (t-test). This figure was drawn using data from reference [33].

Sex differences in blood pressure were observed in animals as early as 1962. Van Liere studied mean arterial pressure (MAP) (i.e., the perfusion pressure exerted on the organs) in 147 adult dogs and found an 8 mm Hg difference in MAP between the males and females [33] (Figure 3). When MAP is graphed as a percent of this dog population by sex, the data reveal a higher percentage of the female population than the male population at the lowest ranges (60-119 mm Hg), while at the highest ranges (160-199 mm Hg), the population was primarily male [33]. The MAP data in this dog population of unknown age mirrors the lower blood pressure observed in normotensive women compared to normotensive men (Figure 1) and the lower prevalence of hypertension in women compared to men in the 18-49 years old age group (Figure 2). Based on the fact that this study investigated blood pressure in street dogs, it is more likely this dog population was comparable to the 18-40 human age group, rather than the 50-70+ human age group, given that street dogs are not likely to realize their full life expectancy due to food constraints, disease, fatal accidents and abuse.

**Figure 4:**
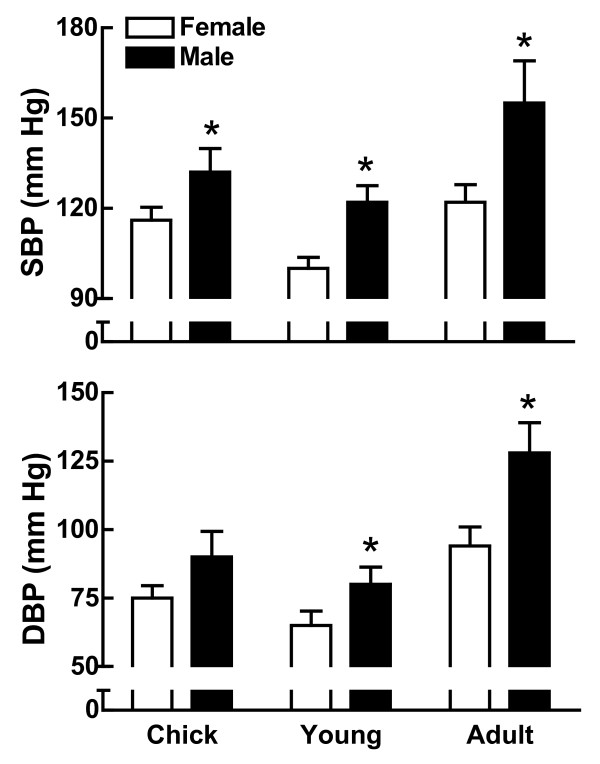
**Blood pressure in male and female chickens across the life span**. Shown is the SBP (top panel) and DBP (bottom panel) in male (black bar) and female (white bar) chickens at different developmental stages from chick (5-7 weeks; n = 7/group), young adult (27-29 weeks; n = 8/group) to full adult (75-80 weeks; n = 6/group). *p < 0.05 vs female, same age group (t-test). This figure was drawn using data from reference [34].

This sex difference in blood pressure is not restricted to the mammalia class of animals (mammals) as it is also observed in Aves (birds). A study of blood pressure from chick to full grown adult in domestic fowl (*Gallus gallus*) showed that male chickens had greater SBP and DBP than the females [34] (Figure 4). This finding in birds is particularly intriguing because sex chromosome dosage in birds is opposite to that found in mammals; the male has two of the same sex chromosomes (designated ZZ in birds) while it is the female that has two different ones (ZW) [1]. What is similar between mammals and birds of the same sex is that their gonadal hormone milieu impacts blood pressure similarly in both animal classes. Birds, therefore serve as a valuable and informative experimental model to investigate the mechanisms underlying sex differences in blood pressure control since they offer unique opportunities to study dosage compensation in the presence of an inverse gonadal hormone milieu from that seen in mammals.

### Sex differences in blood pressure in experimental models of hypertension and the implications for human hypertension

#### Renin-angiotensin system

The renin angiotensin system (**RAS**) plays a crucial role in the control of blood pressure and inhibitors of angiotensin II (**Ang II**) synthesis and Ang II signaling via the angiotensin type 1 receptor (AT_1_R), are widely used to treat hypertension. Ang II infusion is a well studied model of induced hypertension and has been used in rabbits [35], dogs [36], and rodents [37]. In this model, MAP was 20-30 mm Hg higher in males compared to females after Ang II was infused into the inbred C57BL/6J [38] and outbred MF1 [39] mouse strains as well as in Sprague Dawley rats [40]. In fact, there are doses of Ang II in these animals that cause hypertension in the male (defined as a MAP ≥ 135 mm Hg) while leaving the female normotensive. These sex differences can thus be exploited for determining the signaling pathways critical for the development of hypertension and the mechanisms that protect against hypertension.

**Figure 5:**
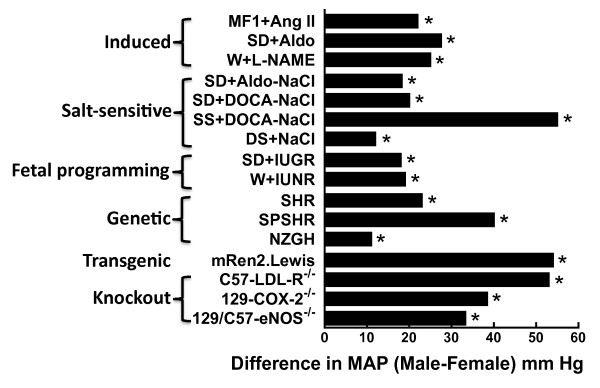
**Sex differences in arterial blood pressure in experimental models of hypertension**. Shown is the difference in arterial blood pressure between males and females in animal models of hypertension including Ang II infusion in MF1 mice (MF1+Ang II) [39]^a^, aldosterone infusion in Sprague Dawley (SD) rats (SD+Aldo) [41]^a ^and L-NAME-treated Wistar rats (W+L-NAME) [44]^c^. Salt-sensitive models include SD rats maintained on a 1% NaCl diet and infused with aldosterone (SD+Aldo-NaCl) [54]^a ^or deoxycorticosterone acetate (SD+DOCA-NaCl) [55]^c ^and, DOCA-NaCl treated Sabra salt-sensitive rats (SS+DOCA-NaCl) [60]^c ^and DS rats maintained on 8% NaCl (DS+NaCl) [57]^a^. Fetal programming models of hypertension include rats subjected to intrauterine growth restriction (SD+IUGR) [64]^a ^and intrauterine nutrition restriction (W+IUNR) [65]^c^, respectively. Genetic models include the SHR [170]^a^, SPSHR [171]^c ^and New Zealand genetically hypertensive (NZGH) rat [172]^c^. Transgenic models include the mRen2.Lewis rat [49]^c^. Single gene knock out models of hypertension include the low density lipoprotein receptor (C57-LDL-R^-/-^) [73]^c^, cyclooxgenase-2 (129-COX-2^-/-^) [74]^c ^and endothelial nitric oxide synthase (129/C57-eNOS^-/-^) [76]^b ^on the C57BL/6, 129Sv and 129Sv/C57BL/6J background strains, respectively. *p < 0.05, male vs female (see cited references for experimental and statistical details of individual studies). Note, studies measuring MAP by radiotelemetry^a ^were preferentially cited over studies using indwelling catheters^b ^or those reporting SBP determined by tail plethysmography^c^.

Aldosterone infusion is another model of induced hypertension that involves the RAS and similarly to the Ang II infusion model, MAP can be increased in male rats (Δ26 mm Hg) under conditions where no significant differences in blood pressure are observed in the females [41] (Figure 5). Full dose response studies for the Ang II- and aldosterone-induced models of hypertension will shed light on whether male-female differences in blood pressure are due to sex differences in the dose that elicits the maximum MAP response (Emax) or in the effective concentration that achieves fifty percent of the maximum response (EC_50_); however, ceiling effects may confound such studies since the frequency of strokes and seizures resulting in the animal's death markedly increases with advancing hypertension.

Nitric oxide plays a fundamental role in the control of blood pressure [42]. Inhibition of its synthesis by chronically administering N^ω^-nitro-L-arginine methyl ester (L-NAME) was shown to cause persistent hypertension [43]. When normotensive Wistar rats were treated with L-NAME, the males exhibited 27 mm Hg higher MAP than their female counterparts [44] (Figure 5). Like the Ang II and aldosterone infusion models, the hypertension induced by inhibiting nitric oxide synthesis can be attenuated by antagonists of the AT_1_R [45] or angiotensin converting enzyme inhibitors [46,47]. The hypertension that develops can even be reversed by inhibiting Ang II synthesis after the hypertension is established [48]. At this point, it is not known whether the RAS plays a permissive or causative role in L-NAME-induced hypertension; however, the finding that all three of these induced models of hypertension are RAS-dependent supports the concept that sex differences in the regulation of the RAS contribute to sex differences in control of arterial blood pressure in these models. In fact, our lab and others have postulated that the lower blood pressure observed in females in RAS-dependent models of hypertension is due to the ability of the female (in comparison to the male) to achieve lower plasma and tissue levels of Ang II (e.g., by increased catabolism of Ang II [39,49]) and/or through maintaining lower numbers of functional AT_1_Rs in the membrane of key target tissues like the kidney [50], which would result in less AT_1_R-mediated vasoconstrictor action in the female compared to the male (see excellent review by Sullivan [51]). These findings of sex differences in the activity of the RAS in experimental animal models of hypertension are supported by clinical studies. Renin is is the rate limiting step in Ang II synthesis and women are reported to have lower prorenin and renin levels than men [52]. Furthemore, an interesting paper by Mirza et al. [53] suggests the combination of E_2 _with an AT_1_R antagonist is more effective than either alone at lowering blood pressure in postmenopausal hypertensive women. These studies highlight the need to fully understand the sex differences in the mechanisms of blood pressure control to achieve optimal treatment regimens for men and women.

#### Diet and blood pressure

As discussed above, blood pressure can also be raised by increasing dietary sodium. In salt-resistant individuals, the increase in MAP may only be a couple of mm Hg, whereas in salt-sensitive individuals, this salt-dependent increase in arterial blood pressure can be substantial [18]. Experimental models of salt-sensitive hypertension include chronic treatment with aldosterone [54] or deoxycorticosterone acetate (DOCA) [55] in the presence of 1% NaCl in the drinking water. In both these induced models, the effect of dietary sodium on MAP was more than 20 mm Hg higher in the male than in the female (Figure 5).

**Figure 6:**
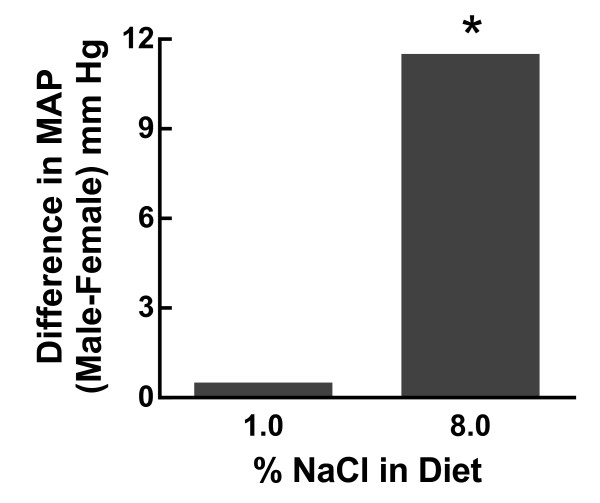
**Effect of dietary sodium on the sex differences in MAP in the DS rat**. Shown is the difference in MAP between males and females in the DS rat as a function of dietary sodium; p < 0.05, male vs female (2-way ANOVA, sex, time). This figure was drawn using data from reference [57].

**Figure 7:**
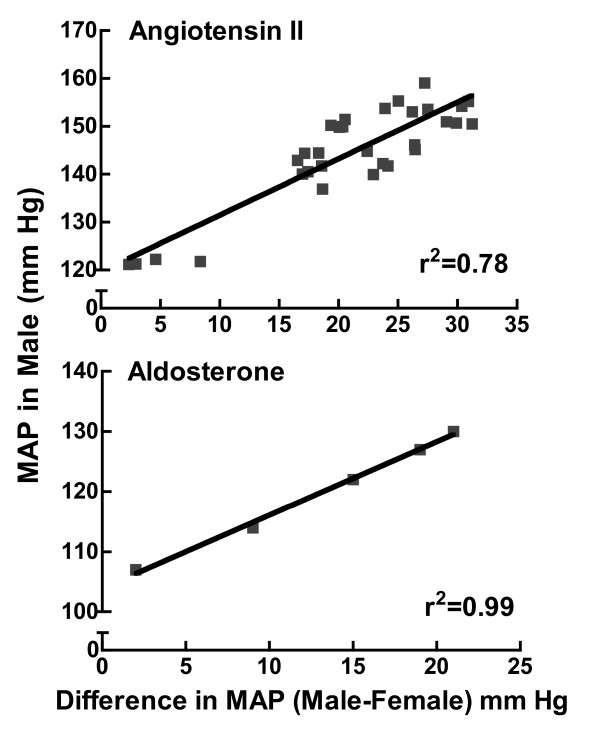
**Relationship between the magnitude of hypertension in Ang II and aldosterone infused males and the sex difference in MAP**. Shown is the MAP in males after angiotensin II (top panel) or aldosterone (bottom panel)-induced hypertension as a function of the sex difference in MAP (male-female, mm Hg). The data and correlation coefficient (r^2^) were drawn using data from references [39,41].

The Dahl salt-sensitive (DS) rat is the most widely studied genetic model of salt-sensitive hypertension and was derived from Sprague Dawley rats by inbreeding based on their susceptibility or resistance to a high sodium chloride (HS) (7% NaCl) diet [56]. Maintained on a low sodium chloride (LS) diet (0.15% NaCl), these animals remain normotensive for many weeks; however, raising the dietary sodium percent by 1-8% induces hypertension in both sexes in a graded manner though to a greater extent in the male than the female [57] (Figure 6). This positive relationship between the sex difference effect size (difference in MAP between male & female) and the magnitude of the hypertension is also observed in the Ang II and aldosterone infusion models (Figure 7). In other words, as the degree of hypertension increases in the male, the magnitude of the sex difference in MAP increases. Some studies suggest that even when blood pressure is identical between males and females, the end organ damage is far greater in the male than in the female. For instance, the JNC7 reports that the age-associated increase in cardiovascular disease risk is markedly greater in men than women at identical SBP and DBP [2]. We have found similar results in the renal wrap model of hypertension. At equivalent MAP, the male rats exhibited far more renal injury than the females as measured by the glomerular sclerotic index, mean glomerular volume, and proteinuria [58]. These clinical and experimental findings suggest sex differences not only exist in the incidence and magnitude of hypertension but also in the adverse effects of hypertension on end organ damage.

At the low end of the Ang II (50 ng/kg/min) dose response curve, a telemetry study conducted in Sprague Dawley rats found that Ang II actually *decreased *MAP in the females but had no effect in the males [59]. This blood pressure lowering effect of low dose Ang II was shown to be mediated by the angiotensin type 2 receptor (AT_2_R) since an antagonist of the AT_2_R prevented this effect. The authors concluded that the lower expression of the AT_2_R in male target tissues (e.g., the vasculature and kidney) compared to the female made males less able to realize the benefits of the AT_2_R-mediated blood pressure lowering effects. These interesting findings emphasize the critical importance of taking into account the sex of the animal when investigating physiological mechanisms of AT_2_R action. Furthermore, this study demonstrates how mechanisms modulating blood pressure in one sex can not necessarily be extrapolated to the opposite sex.

The Sabra salt-sensitive (SS) rat was developed using a similar strategy to that used to derive the DS rat [60]; however, instead of a HS diet, the SS rat was selected based on susceptibility or resistance to DOCA-salt. Similarly to the DS rats, the males have nearly 30 mm Hg higher MAP than the females (Figure 5). These findings of sex differences in salt-sensitivity are also seen in humans. Wilson et al. [61] examined blood pressure responses to changes in dietary sodium in 135 male and female adolescents and found that the girls lowered their blood pressure to a greater degree than the boys after dietary sodium was reduced. Furthermore, when dietary sodium was increased, blood pressure was raised to a greater extent in the boys.

It is not just sodium in the diet that adversely impacts blood pressure, high fat [62] and high fructose [63] diets are also associated with increased MAP. In both cases, the female is protected from the diet-induced hypertension compared to the male. Adverse fetal environments (also known as fetal programming) can induce hypertension in offspring. Intrauterine growth restriction induced by placental insufficiency in late gestation results in hypertensive male offspring; at 12 weeks of age, the MAP reached 158 mm Hg in the males, a 23 mm Hg increase over the control offspring, whereas the blood pressure in the females marginally increased by 13 mm Hg [64] (Figure 5). Intrauterine nutrition deficiency, a model in which the pregnant dams are fed 50% of a normal dietary caloric intake during the gestational period, result in marked hypertension in the male offspring (155 mm Hg SBP) whereas the females become only mildly hypertensive (135 mm Hg SBP) [65] (Figure 5).

The spontaneously hypertensive rat (SHR) was established in the 1960s by selectively breeding Wistar-Kyoto rats with high blood pressure [66] and is currently the most widely studied model of primary hypertension [67]. The stroke prone SHR (SPSHR) was developed using a similar strategy with the prevalence of stroke used to select the strain [68]. In both the SHR [69] and the SPSHR [70], the males develop hypertension at earlier ages than the females and the severity of the hypertension is greater in the males (Figure 5).

The higher blood pressure observed in the males is also found in genetic models of hypertension that over express or under express a specific gene. Over expression of the mouse Ren-2 gene in various rat tissues was the first example of a transgenic model of hypertension [71]. These (mRen2)27 rats exhibit fulminating hypertension that is lethal in the homozygous state if not treated with antihypertensive medications. When these rats were backcrossed onto the Lewis inbred strain creating the congenic mRen2.Lewis rat, the hypertension was less severe, the rats survived and sex differences in MAP became apparent [72] (Figure 5).

For the most part, the factor of sex has been poorly investigated in gene knockout (KO) models of hypertension. In the ones that have measured blood pressure in both sexes, arterial pressure was higher in the males than the females including mice deficient in the low density lipoprotein receptor [73], cyclooxygenase-2 [74], the α1 subunit of soluble guanylate cyclase (sGCα1^-/-^) [75] and endothelial nitric oxide synthase [76]. Unfortunately, blood pressure has only been reported for males in hypertensive mice lacking atrial natriuretic factor, which leads to salt-sensitive hypertension [77] and mice lacking the atrial natriuretic type A receptor, which are hypertensive but not salt-sensitive [78]. Further missed opportunities for understanding sex differences in hypertension include mice lacking the CXC chemokine receptor 3 [79] and 11β-hydroxysteroid dehydrogenase type 2 [80] since only male blood pressure has been reported thus far.

Studying sex differences in these various experimental models of hypertension could provide valuable clues into the intrinsic mechanisms that govern sex differences in blood pressure. For instance, if a sex difference in blood pressure was not found in a particular KO animal after hypertension was induced, then that specific gene would be implicated in the mechanisms contributing to sex differences in that model of hypertension. The corollary experiment implicating a specific gene in the sex differences in hypertension would include mice over-expressing a candidate gene.

### Blood pressure measurement procedures: Implications for the study of sex differences

Radiotelemetry is considered the gold standard for measuring blood pressure in animals [81]. A radiotransmitter is installed near the aorta, which enables MAP to be followed continuously both day and night in a conscious freely moving animal from minutes to several months without the stress associated with restraint [82]. This automatic feature of radiotelemetry is particularly appreciated when studying blood pressure in rodents since rodents spend their normal waking hours during the night. Blood pressure can also be inexpensively measured by tail-cuff plethysmography, which involves placing the mouse or rat in a tube and measuring the blood pressure in the extended tail; however, this restraint can stress the animal [83]. Moreover, the tail-cuff procedure frequently involves heating the animal to increase blood flow in the tail artery, which exerts additional stress as well as introduces a measurement artifact across subjects [84].

The stress component of tail-cuff measurements including immobilization and heat can raise blood pressure. A study in normotensive WKY and hypertensive SHR showed that SBP was approximately 30 mm Hg higher when measured by tail-cuff [WKY, 162 ± 5; SHR, 207 ± 5, mm Hg] than by telemetry [WKY, 131 ± 1; SHR, 178 ± 6, mm Hg] [85]. A study in prenatally malnourished rats showed that the blood pressure increase induced by a noxious odor was augmented by the stress of the tail-cuff procedure itself [86]. The stress from tail-cuff can also affect the response to drugs. Hydralazine had an approximately 2-fold greater hypotensive effect on the SBP when measured by tail-cuff compared to telemetry, indicating that the antihypertensive effects of some drugs may be influenced by the level of stress the animal is experiencing [86]. Sullivan et al. [87] showed that female endothelin B receptor-deficient rats maintained on a HS (8%) diet for two weeks had 12 mm Hg higher SBP than males when measured by tail-cuff; however, in a later study when radiotelemetry was used to measure MAP in these animals, the females no longer had higher blood pressure than the males (David M. Pollock, personal communication). The authors then found that the females exhibited a greater rise in blood pressure in response to acute air jet stress compared to the males, suggesting that in endothelin B receptor-deficient rats, the females were more responsive than the males to acute stress. These findings suggest that the higher arterial pressures observed in the female rats detected by tail-cuff was due to their greater responsivity to the stress of immobilization compared to the male rats.

Blood pressure can also be measured under anesthesia using carotid arterial cannulation; however, blood pressure measured under anesthesia is typically 10-20 mm Hg lower than when measured in conscious freely moving animals [88,89]. This observation demonstrates that anesthesia in and of itself can affect arterial pressure and thus differential responses by males and females to anesthetics [90] could confound interpretation of blood pressure studies between the sexes.

In people, blood pressure is typically measured using a stethoscope and sphygmomanometer after an individual has been sitting calmly for a few minutes because blood pressure can be elevated by physical activity and emotional stress. In fact, there is a documented phenomenon called "white coat hypertension", which is defined as a normotensive individual who exhibits increased SBP and DBP of at least 20 mm Hg and 10 mm Hg, respectively, in the presence of a health care provider. Multivariate analysis of 5,716 patients in Ireland over a 22 year period demonstrated that females have a higher prevalence of white coat hypertension than males [91]. A higher prevalence of white coat hypertension in women was also observed in 2,462 patients in Morocco [92] and in a Korean population of 967 patients [93]. White coat hypertension is associated with increased sympathetic activity [94,95], decreased parasympathetic-modulation [94,96], increased activity of the RAS [95], higher scores on measures of conditioned anxiety [97] and higher levels of adrenocorticotrophic hormone and cortisol after challenge with corticotropin releasing hormone [98]. Together, these data suggest that arterial pressure in women exhibits more hypothalamic-pituitary-adrenal hypersensitivity to stressors than arterial pressure in men.

### Sex differences in the genetic basis for salt-sensitive hypertension

**Figure 8:**
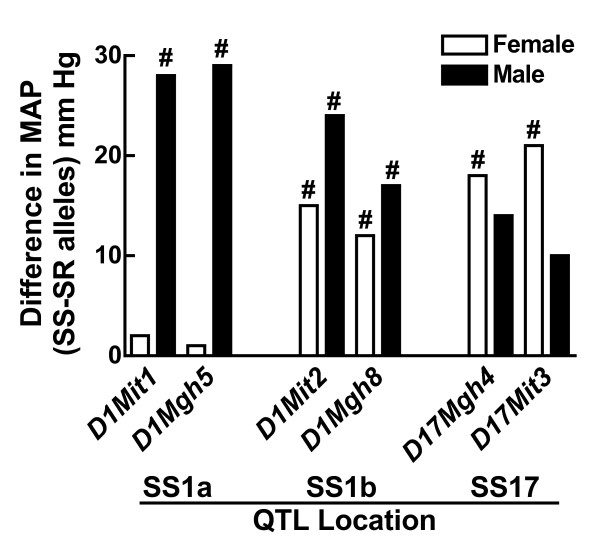
**Sex differences in QTL locations of salt-sensitive hypertension in the SS rat**. Shown is the difference in MAP between the SS rat and rats in which SS alleles were introgressed into female (white bar) and male (black bar) salt-resistant rats (SR alleles) at the SS1a, SS1b and SS17 QTL locations. #p < 0.05 vs SS, same sex. This figure was drawn using data from reference [99].

Sex differences in the genetic basis for salt-sensitivity was observed in the Sabra salt-sensitive (SS) rat by linkage analysis in segregating populations derived from the SS (a.k.a. SBH/y) and Sabra salt-resistant strain (SR a.k.a. SBN/y). Whereas two quantitative trait loci (QTL) for salt-sensitive blood pressure were found on chromosome 1 in the male (*SS1a and SS1b*), in the female only the *SS1b *was found to be a QTL [99] (Figure 8). Moreover, a QTL on the X chromosome was identified only in the female [100]. Consomics were then constructed in which these two QTL on chromosome 1 from the SR rat were introgressed into the SS genetic background; however, these studies failed to show sex differences in the QTL [60].

**Figure 9:**
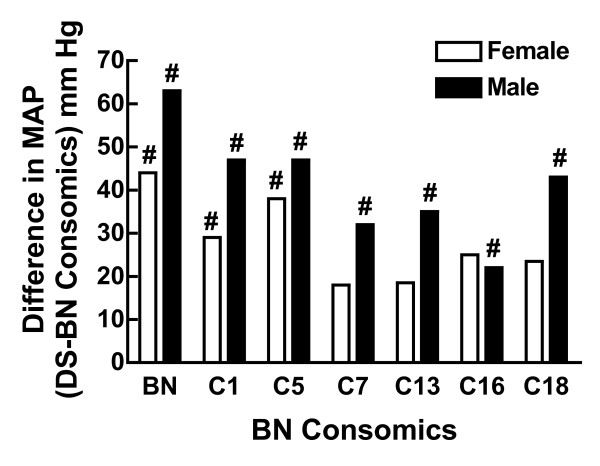
**Sex differences in the chromosome location of salt-sensitive hypertension in the DS rat**. Shown is the difference in MAP between the DS rat and the salt-resistant Brown Norway (BN) rat or DS consomics in which chromosomes (C) 1, 5, 7, 13, 16 and 18 were substituted with the respective BN chromosomes in male (black bar) and female (white bar) rats. Note that the maximum difference in salt-sensitivity is expected to be between the DS and BN rats. #p < 0.05 vs DS, same sex. This figure was drawn using data from reference [101].

Consomics have also been used to study the genetic basis of hypertension in the DS rat. Twenty two consomic strains were generated in which individual chromosomes from the Brown Norway salt-resistant rat were introgressed into the DS (SS/Mcwi) genetic background. MAP was measured in both male and female rats fed a high-salt (8.0% NaCl) diet for 3 weeks, and was found to be significantly greater in the male and female (177 ± 3 and 160 ± 7 mmHg, respectively) SS/Mcwi rat than in the male and female (111 ± 1 and 112 ± 3 mmHg, respectively) Brown Norway rats. Similarly, MAP was higher in males than females in certain consomic strains. Mattson et al. [101] found that substitution of chromosomes 1, 5, 7, 13, 16 and 18 from the Brown Norway onto the DS background attenuated the development of hypertension in male rats (Figure 9). In female rats, substitution of chromosomes 1 and 5 also decreased blood pressure but not chromosomes 7, 13, 16 or 18 (Figure 8). These findings demonstrate that the genetic basis of salt-sensitivity in the DS rat is distinctly different between males and females and this research also emphasizes the value of consomics for dissecting the genetic basis of sex differences in the control of blood pressure.

### Effect of gonadal hormones on blood pressure

#### Ovaries and 17β-estradiol

It has been difficult to disentangle the effects of ovarian function on arterial pressure in women from those of aging because ovarian hormone loss is an age-associated event. Cross-sectional studies (Figures 1 &2) suggest the rate of the age-associated rise in arterial pressure increases around the onset of menopause (i.e., 51 years of age) and prospective population studies show that postmenopausal women have higher arterial pressure than age-matched premenopausal women [102-104]; however, inferences from these studies are limited because the populations within the groups compared are not likely to differ solely on the basis of age of menopause onset.

Comparing arterial pressure between young premenopausal women and women with premature ovarian failure provides another way to assess ovarian hormone loss on blood pressure since premature ovarian failure typically presents at 27 years of age. Women with premature ovarian failure have a higher incidence of hypertension than age-matched premenopausal women suggesting ovarian hormone loss raises arterial pressure independently of the effect of aging on blood pressure. These studies, however, are confounded by the possibility that premature ovarian failure represents an acceleration of the aging process (see review by Pal & Santoro [105]).

Turner syndrome provides a window into the blood pressure effects of ovarian function at an even earlier age since girls with Turner syndrome exhibit ovarian hormone deficiency during puberty. Compared to published population standards in girls, girls with Turner syndrome have higher blood pressure, a higher incidence of abnormal blood pressure circadian rhythms, and a higher incidence of idiopathic hypertension even in the absence of aortic coarctation, renal abnormalities or chronic urinary track infections [106,107]. Turner girls do not have a complete double X chromosomal complement. Therefore, comparing arterial pressures in girls with and without Turner syndrome is confounded by sex chromosome complement differences.

Longitudinal studies investigating the relationship between arterial pressure and the age of menopause onset have not demonstrated an effect of menopause on arterial pressure that is distinct from the effect of aging. Hjortland et al. [108] conducted a 5 year longitudinal study in a cohort of 1686 women who were 40-41 years old. van Beresteyn et al. [109] followed 193 perimenopausal women between 49 to 56 years of age for over 10 years and Mathews [110] examined blood pressure in 541 healthy premenopausal women 42-50 years of age for approximately 2 & 1/2 years. While none of these longitudinal studies detected an effect of menopause on arterial pressure that was distinct from aging, following changes in blood pressure in women longitudinally could have been confounded by antihypertensive medications since it is unethical to withhold antihypertensive medicine in hypertensive subjects. While it is clear that arterial blood pressure increases both with aging and ovarian hormone dysfunction in women, it is not known to what extent the mechanistic pathways of these two hypertension-associated factors overlap. Increased salt-sensitivity may be a mechanism that contributes to postmenopausal hypertension; however, whether or not postmenopausal women are more salt-sensitive than premenopausal women remains unresolved. As noted above, it is difficult to tease out age effects in women from effects due to changes in the ovarian hormonal milieu; however, much can be learned from experimental animal models.

**Figure 10:**
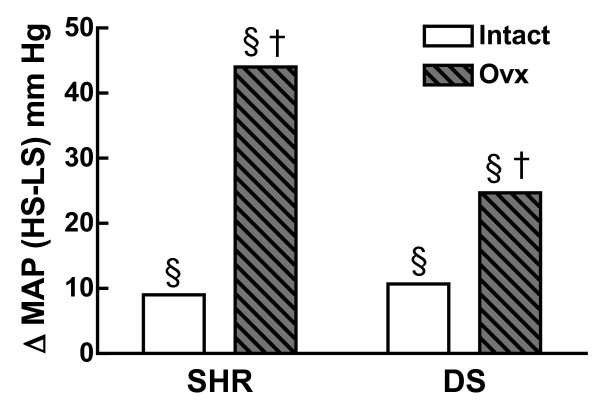
**Effect of ovariectomy on salt-sensitivity**. Shown is the difference in MAP between HS (8% NaCl) and LS diets 0.15-0.60% NaCl in the intact (white bar) and ovariectomized (Ovx) (striped bar) SHR [112] and DS rat [57]. §p < 0.05 vs Intact, same rat strain, same dietary sodium; †p < 0.05, HS vs LS, same rat strain, same gonadal state (see cited references for experimental and statistical details of individual studies).

The mRen2.Lewis rat exhibits salt-sensitive hypertension, which was not found to be magnified by ovariectomy [111]; however, SBP was measured by tail-cuff in this study and thus, the possibility remains that restraint stress due to the measurement method confounded these findings. Furthermore, the SBP is so high in the ovariectomized mRen2.Lewis rat maintained on a HS diet for 15 weeks (approximately 230 mm Hg) that the SBP may have reached a ceiling effect (i.e., the SBP just couldn't get any higher without the animal's demise). The effects of ovariectomy on salt-sensitivity have also been examined in the SHR [112] and DS rat [57]. In these models, the salt-sensitive component (ΔMAP HS-LS) was found to be amplified by ovariectomy (Figure 10) indicating that the loss of ovarian hormones increases salt-sensitivity. It will be important to determine how similar these findings are to other animal models of salt-sensitivity.

**Figure 11:**
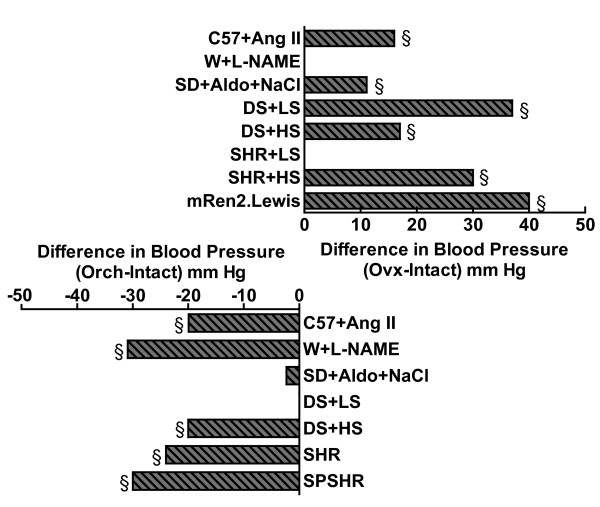
**Effect of gonadectomy on blood pressure in experimental models of hypertension**. *Top panel*, Shown is the difference in blood pressure between ovariectomized (Ovx) and intact (Intact) females in animal models of hypertension including Ang II infusion in C57 mice (C57+Ang II) [38]^a^, Wistar rats infused with L-NAME (W+L-NAME) [44]^c^, SD rats treated with deocycorticosterone acetate and HS (SD+DOCA+NaCl) [54]^a^, DS rats [57]^a ^and the SHR [112]^a ^maintained on a LS and HS diet and the mRen2.Lewis rat [111]^c^. *Bottom panel*, Shown is the difference in blood pressure between orchiectomy (Orch) and intact (Intact) males in animal models of hypertension including C57+Ang II [38]^a^, W+L-NAME [44]^c^, SD+DOCA+NaCl [54]^a^, DS maintained on a LS and HS diet [173]^a^, SHR [146]^b ^and the SPSHR [145]^c^. §p < 0.05 vs Intact, same sex (see cited references for experimental and statistical details of individual studies). Note, studies measuring MAP by radiotelemetry^a ^were preferentially cited over studies using indwelling catheters^b ^or those reporting SBP determined by tail plethysmography^c^.

Much can be learned by comparing models in which ovariectomy had and did not have an effect on arterial pressure. While ovariectomy increased arterial pressure in several animal models of hypertension including Ang II- [38,39] and DOCA- [54] infusion and the DS [113] and mRen2.Lewis [111] rats (Figure 11), ovariectomy did not raise arterial pressure in the SHR on a normal sodium chloride diet [112] or the Wistar rat treated with the nitric oxide synthase inhibitor, L-NAME [44]. For instance, does the finding that ovariectomy has no effect on arterial pressure in the L-NAME Wistar model suggest that ovariectomy elicits its arterial pressure raising effects by reducing nitric oxide in key target tissues such as the kidney? This possibility could be addressed by comparing bioavailable nitric oxide levels and activity in the kidneys in these various models before and after ovariectomy. Taken together, these studies indicate that the presence or absence of ovarian hormones is not the only factor that underlies sex differences in blood pressure given the mixed findings regarding the effect of ovariectomy on arterial pressure (Figure 5).

To address ovarian hormone regulation of blood pressure, most investigators have compared intact animals with ovariectomized animals treated with and without 17β-estradiol (E_2_) treatment. E_2 _treatment prevented the blood pressure raising effects of ovariectomy in the Ang II- [114] and DOCA- [54,115] infused models as well as in the mRen2.Lewis rat, the DS rat on both LS [82] and HS [116] diets and the SHR on a HS diet [112]. The consistency of the ability of E_2 _to prevent the blood pressure raising effects of ovariectomy across these distinct experimental models of hypertension, suggests the mechanisms by which E_2 _loss raises blood pressure are robust and highly conserved and thus support the conclusion of cross-sectional studies showing that menopause increases arterial pressure in women.

Most experimental studies of E_2 _depletion and replacement on arterial pressure have been conducted in young animals. Therefore, it has been difficult to isolate effects of E_2 _deficiency from aging effects. Hinojosa Laborde et al. [82] addressed this question by following MAP by telemetry for a year in DS rats maintained on a LS diet. This study found that ovariectomy at a young age accelerated the age-associated increase in MAP whereas E_2 _replacement in the ovariectomized rats markedly attenuated this effect. The fact that MAP was significantly lower in the E_2 _replaced group compared to the intact group after one year suggests E_2 _treatment can protect against the age-associated increase in MAP [82]. At 1 year of age, plasma E_2 _in the ovariectomized DS rats treated chronically with E_2 _was 33% < levels found in young 4 mo DS rats [82]. Therefore, it would be informative to modify the E_2 _replacement regimen in the ovariectomized rats so that similar E_2 _levels to the young rats were achieved even after 1 year and then determine if these higher plasma E_2 _levels further improved upon or even totally prevented the age-associated increase in MAP. Mimicking the cycling of E_2 _that is found in the young intact female is another important variable that needs to be investigated. A constant replacement dose of E_2 _might desensitize ERs in a manner similar to how super agonists of the leutinizing hormone (LH) receptor cause receptor down regulation [117]. Thus, it is possible that mimicking the estrus cycle would result in greater attenuation of the age-associated increase in arterial pressure by preventing chronic ER desensitization.

Far less is known regarding the effects of progesterone loss on blood pressure since few studies have investigated the role of ovarian hormones other than E_2_. In 1997, Crofton et al. [115] found that progesterone had no effect in and of itself on SBP in ovariectomized rats in the DOCA-NaCl model; however, this study did find that progesterone slowed the ability of E_2 _treatment to attenuate the blood pressure raising effects of ovariectomy. Studies are clearly needed to investigate the role of progesterone in diverse experimental models of hypertension to fully understand the role of progestins in blood pressure control. This is particularly relevant to women on hormone replacement regimens. In fact, clinical studies have shown that some progestins impair endothelial function in women while others do not, suggesting that certain progestins like depot-medroxyprogesterone acetate can inhibit the beneficial effects of E_2 _while others like drospirenone are without effect [118-120].

Ten years ago, studies of estrogen receptor alpha and beta (ERα & ERβ) null mice suggested that ERβ protects against the age-associated increase in arterial pressure since at 6-7 months of age compared to wild type (WT), SBP was shown to be elevated by 17 and 28 mm Hg in the female and male KO mice, respectively [121]. In contrast, the ERα null mouse did not show a similar age-associated increase in SBP. Pharmacological studies using ER subtype selective ligands in the SHR revealed that ligand-dependent activation of ERβ lowers arterial pressure to a greater extent than E_2 _alone or the ERα agonist [122]; however, these studies were conducted solely in the female so this study did not assess if sex differences in the activity of these receptors contributes to sex differences in arterial pressure.

The gonadal hormone milieu changes markedly throughout the life of a woman. After puberty, E_2 _levels markedly increase by 5-10-fold [123,124]. Five years or more after menopause, E_2 _levels drop by greater than 10-fold compared to levels in normal cycling women [124]. The vast majority of research in menopause [125] has centered on the role of the precipitous drop in E_2 _secretion from the ovarian follicles that occurs at this life transition [126]; however, other significant hormonal changes are also occurring. By the onset of menopause, T declines by approximately 40% [124]. The fact that the decline in T is far less dramatic than the drop in E_2 _means that the T:E_2 _ratio markedly increases as a woman transitions into menopause.

As women approach menopause, atresia of the ovarian follicles occurs, fertility declines and serum follicle stimulating hormone (FSH) and LH start to surge in an effort to produce more ovarian follicles [126]. After menopause, there is more than a 10-fold increase in the levels of FSH and LH, the pituitary hormones of the hypothalamic-pituitary-ovarian axis, that are secreted in response to the hypothalamic hormone, gonadotropin releasing hormone [124]. The secretion of these hormones is tightly regulated through hypothalamic-pituitary-ovarian feedback mechanisms. Supplementation with E_2 _markedly reduces FSH and LH levels in postmenopausal women, although levels remain significantly higher than those found in premenopausal women [126]. Studies investigating the role of E_2 _in the age-associated increases in arterial pressure are confounded by changes in these hormones of the hypothalamic-pituitary-ovarian axis and therefore, the effects of E_2 _deficiency can not be separated from the effects of rising FSH and LH levels.

Some studies suggest that elevation of FSH contributes to hypertension-associated disease. Chu et al. [127] showed that elevated FSH in premenopausal cycling women is associated with increased cardiovascular risk. The authors studied 40 women between the ages of 29 to 49 with normal menstrual cycles and premenopausal levels of E_2 _who were not receiving exogenous hormone or statin treatment. Total cholesterol and low density lipoprotein were significantly higher in women who had an FSH level ≥ 7 IU/I compared to those with an FSH ≤ 7 IU/I and these effects were *independent *of age. Patients with low FSH had E_2 _levels around 27 pg/ml whereas patients with high FSH had E_2 _levels around 52 pg/ml. If the increased prevalence of cardiovascular risk factors is solely due to lower E_2 _levels then the opposite findings would be expected, namely, that the premenopausal women with higher plasma E_2 _levels would have less cardiovascular risk factors than those women with lower plasma E_2 _levels. This study implicates a role for FSH and LH in and of themselves in cardiovascular risk.

Inactivating mutations in the FSH receptor gene have been reported to cause hereditary hypergonadotropic ovarian failure in women [128]. More recently, Nakayama et al. [129] examined 5 single nucleotide polymorphisms in the FSH receptor gene in more than 1000 essential hypertension patients and age-matched controls in a subgroup analysis of the Hypertensive Section of the Japanese Millennium Project. One single nucleotide polymorphism in the 5'-untranslated region of the FSH receptor gene occurred with increased frequency in women with hypertension. Patients with the A/A genotype in this polymorphism exhibited lower levels of FSH receptor transcriptional activity and had lower E_2 _levels than those without the A/A genotype (G/G or G/A). These studies suggest that this single nucleotide polymorphism is a susceptibility mutation for essential hypertension in women and underscores the value of future studies that focus on ovarian hormone changes in addition to E_2 _in blood pressure control. Surprisingly, the roles of T, LH and FSH and changes in their ratio to E_2 _(e.g., T:E_2_, LH:E_2 _& FSH:E_2_) throughout a woman's life span are generally neglected in hypertension research and consequently, much less is known regarding their specific effects on blood pressure control.

#### Testes and testosterone

There are fewer studies investigating the influence of the testicular hormonal milieu on blood pressure compared to studies of the ovarian hormonal milieu. As in women, as men age, the prevalence of hypertension increases (Figures 1 &2). Accompanying this rise in arterial pressure is a gradual decrease in total T. By the time men reach the eighth decade, free T drops to approximately 50% of the lifetime maximum [130-133]. This gradual decrease in T is misleading since bioavailable T constitutes only a small fraction (1-3%) of serum T since the majority of this circulating steroid is bound to sex-hormone binding globulin [134,135]. Since sex-hormone binding globulin levels increase nearly 2-fold over the male lifespan, bioavailable T is markedly decreased by aging [136,137]. A positive correlation among aging, arterial pressure and decreasing bioavailable T exists and hypertensive men have lower T [138-140] and androstenedione [140] than normotensive men. This inverse relationship between T and arterial pressure [141] suggests that hypertension in aging men is associated with decreased T activity.

While it is difficult to isolate the effects of declining T levels on blood pressure from other effects of advancing age on hypertension-associated disease [142], studies of oncology patients support the idea that T deficiency increases blood pressure in men. Surgical removal of the testes due to testicular cancer results in higher arterial pressures than that found in age-matched controls [143]. Furthermore, there was an inverse association between T levels and arterial pressure in these cancer survivors. Thus, these studies suggest that T deficiency has an adverse effect on arterial pressure. A study of 22 prostate cancer patients found that central arterial pressure was increased after T deficiency was induced by 3 mo treatment with LH-releasing hormone agonists [144]. Since T is converted to E_2_, lower levels of T in men are also associated with lower levels of E_2_. LH receptor agonists not only reduced T from 14.5 to 1.2 nmol/liter, E_2 _was reduced by 3-fold [144]. Thus, it remains unclear as to whether the increased arterial pressure in these men was due to the loss of T, decreased E_2 _or changes in the T:E_2 _ratio.

In contrast to the clinical data suggesting that T deficiency exacerbates hypertension, T deficiency induced by orchiectomy lowers blood pressure in several experimental models of hypertension including Ang II infusion [38], DOCA-salt [54], chronic L-NAME treatment [44] and in the SPSHR [145] and SHR on either a normal salt and HS diet [146]. Orchiectomy also reduced MAP by 33 mm Hg in the α1 soluble guanylate cyclase KO mouse [75]. In contrast, orchiectomy had no effect on DS rats maintained on a LS diet although on a HS diet, removing the testes reduced arterial pressure in these animals [116] (Figure 11). What makes the DS-LS model different from these above models is that the DS-LS rats were normotensive. Thus, it is worth exploring whether or not testicular hormones only affect blood pressure in hypertensive models. Investigators have compared intact with orchiectomized animals treated with and without T. Treatment with T prevented the arterial pressure lowering effects of orchiectomy in the DOCA-NaCl [115] infused model as well as in the SHR [69,146-148], suggesting that T deficiency due to the gonadectomy lowers arterial pressure in these models of hypertension.

The absence of the androgen receptor (AR) did not affect arterial pressure under basal conditions as no differences in MAP were found between male WT and AR null mice (ARKO) nor was there any effect of AR deficiency on the magnitude of Ang II-induced hypertension [149]. This study suggests the AR is not a major factor in the control of arterial blood pressure. In contrast to conclusions drawn from comparing male ARKO and WT mice, the AR antagonist flutamide reduced blood pressure in the male SHR [145,150] and the male SPSHR [145]. Flutamide also attenuated the development of hypertension in male TGR(mRen2)27 rats [151] and the male α1 soluble guanylate cyclase KO mouse [75]. These studies with flutamide indicate that inhibiting the AR lowers arterial pressure suggesting that the AR contributes to hypertension. To address the discrepancy between the ARKO mouse data and the flutamide data, experiments are needed to ensure flutamide is acting solely through the AR and that no pharmacological differences exist in the Emax or EC_50 _in the Ang II-blood pressure dose response curve between WT and ARKO mice.

The cytochrome P450 enzyme aromatase produces E_2 _and mice with a targeted disruption of the aromatase gene are deficient in E_2_. No detectable differences in SBP were reported between the aromatase KO and WT female mice [152]. The finding that a deficiency in E_2 _induced by aromatase disruption had no detectable effect on SBP is consistent with studies in WT mice that showed E_2 _depletion by ovariectomy did not alter basal arterial pressure [38,39] and experiments in female rats that showed aromatase inhibition did not alter basal arterial pressure [153]. The aromatase KO did however exhibit a lower basal DBP than the WT and this was ascribed to greater BP variability within mid and high frequency bands and loss of autonomic control of the heart. Although the authors did not induce hypertension in the aromatase KO mice, one might predict that the KO females would have a higher magnitude of Ang II-dependent hypertension than their WT littermates since ovariectomized mice have a higher degree of Ang II-dependent hypertension than the intact females [38,39]. These are important experiments to conduct since previous studies with the aromatase inhibitor 10-propargyl-androst-4-ene,3,17-dione (19-AA) showed that 19-AA attenuated arterial pressure in an inbred salt-sensitive rat strain [154] and the SHR [155]; however, 19-AA also inhibits non-aromatizing adrenal 19-hydroxylation - a key step in forming 19-nordeoxycorticosterone, so it was unclear whether or not the attenuation of BP was due to E_2 _deficiency or lowering of 10-nor-corticosteroids in these experiments.

Why then does this majority of the animal data directly conflict with the clinical data showing T deficiency is associated with blood pressure elevation? One explanation is the discrepancy in age. Animal castrations for the most part were performed on young animals while the clinical findings are primarily reported on men in their fifth to seventh decades of life. In fact, when orchiectomies in the SHR were conducted at 6 mo of age [145], there was no effect of the gonadectomy on SBP whereas when castration was performed between 3 weeks to 4 months [69,146-148], orchiectomy markedly attenuated the magnitude and slowed the rate of the developing hypertension. Taken together, these clinical and experimental studies suggest that T has bimodal effects. At high levels such as found at early ages, T adversely contributes to mechanisms of hypertension, while at later ages when T levels naturally decline, there is insufficient T protection. Perhaps T action follows a physiological U-shaped response in which doses that are too low or too high are equally disadvantageous to the animal.

Studies of T and blood pressure have also been investigated in the female. In women with excess T production, T has a blood pressure elevating effect. Women with polycystic ovary syndrome have elevated T levels and there is a positive association between T levels and arterial pressure [156]. This latter observation emphasizes that T dosage or ratio to other hormones (e.g., T:E_2_) may impact blood pressure. Clearly more research into of T action is needed to sort out the role of T, E_2 _and the ratio of T:E_2 _in blood pressure control in both the male and female.

### Effect of the sex chromosomes on blood pressure

Although gonadal sex is a major difference between men and women, it is not the only difference. The sex chromosome complement is also distinct. Men have one X and one Y chromosome whereas women have two X and no Y chromosomes. Thus, the sex chromosome gene dosage is different between the sexes. Men express Y chromosome genes that women do not. Moreover, the second X chromosome is inactivated in each cell in the woman's body, but this inactivation is not complete and 15-17% of genes on the inactivated X chromosome (approximately 200-300 genes) escape X-inactivation [157]. Therefore, the physiology of blood pressure control could be differentially affected by sexual dimorphism in the expression of these X-linked genes. Furthermore, men only express the X from their mother (X^m^) while women express 50% of their X genes from their father (X^p^) and 50% from their mother (X^m^). Thus, men and women possess distinct differences in parental imprinting of the X chromosome which can have a profound effect on physiology and pathophysiology [158].

There are many men walking around with more than one Y chromosome, only they don't know it because it is rarely associated with a phenotype except perhaps that of being taller than usual. These XYY men have normal blood pressure, suggesting Y gene dosage does not adversely affect blood pressure [159]. In contrast, women with XO Turner syndrome exhibit a higher incidence of hypertension than age-matched women with premature ovarian failure (46, XX) [160]. Thus, the loss of the second X negatively impacts arterial pressure, suggesting loss of gene expression from the second X has significant consequences for normal blood pressure control. A study comparing the lipid profile in X^m^O versus X^p^O Turner women suggests maternal imprinting of the X chromosome adversely affects the lipid profile compared to paternal imprinting [161]. Triglycerides, low density lipoprotein, cholesterol and visceral body fat content were all higher in X^m^O compared to X^p^O. While blood pressure was not investigated in this study, the association between hypertension and adverse lipid profiles, suggests parental imprinting impacts blood pressure control as well.

**Figure 12:**
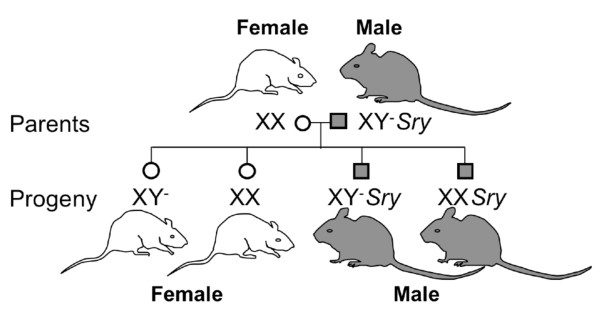
**Generation of the four core genotype**. The *Sry *gene was spontaneously deleted from the Y chromosome (Y^-^) and transferred to an autosome resulting in the XY^-^*Sry *male mouse. Breeding XX females with XY^-^*Sry *males produces the four core genotype: XY^- ^and XX females and XY^-^*Sry *and XX*Sry *males. We refer to XX and XY^- ^as XX and XY-Females and XX*Sry *and XY^-^*Sry *as XX- and XY-Males throughout the text.

Studies in the four core genotype mouse model are well suited for investigating mechanisms between the sexes since sex chromosome effects can be distinguished from gonadal sex effects (Figure 12). In this model, the *Sry *gene was deleted from the Y chromosome through a natural mutation (Y^-^) [162]. Thus, the XY^- ^mouse does not develop testes, but instead, develops ovaries and expresses a female gonadal hormone phenotype. The *Sry *gene was also inserted onto an autosome, creating XY^-^*Sry *and XX*Sry *transgenic mice that regardless of the sex chromosome complement (XY vs. XX) are gonadal males (see review by Arnold [163]).

**Figure 13:**
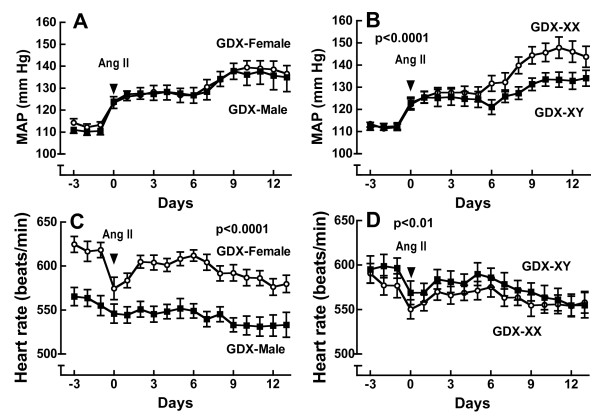
**MAP and HR after Ang II infusion in GDX four core genotype mice**. Shown are the mean ± SEM for MAP **(A) **and HR **(B) **two weeks after Ang II infusion at 200 ng/kg/min in the GDX four core genotype; XX-Female (white, n = 9), XY-Female (horizontal stripes, n = 9), XX-Male (diagonal stripes, n = 8) and XY-Male (black, n = 8). Two-Way ANOVA showed that the sex chromosomes account for 16% of the total variance (*p < 0.03 vs. XX, regardless of gonadal sex) on MAP whereas there was no gonadal sex effect (p = 0.58) nor any interaction between the sex chromosomes and gonadal sex (p = 0.90). There was no SCE (p = 0.55) or interaction between the sex chromosomes and gonadal sex (p = 0.13) on HR. Gonadal sex accounted for 31% of the total variance (#p < 0.001 vs female, regardless of sex chromosome complement) in HR. This data is republished from reference [39].

We have used the four core genotype mouse model to isolate sex chromosome effects from gonadal sex effects in the Ang II-induced model of hypertension. We found that the magnitude of the hypertension was greater in gonadectomized XX mice compared to gonadectomized XY mice regardless of whether the mice were born male or female [39] (Figure 13). This finding suggests that under conditions of gonadal hormone deficiency, a sex chromosome effect is either unmasked or expressed. We do not know at this point whether the sex chromosome effect on MAP is due to the XX or XY sex chromosome complement. If it is housed in the XX complement, then this finding may have negative implications for women with ovarian hormone deficiency such as women with premature ovarian failure or postmenopausal women. In this case, an adverse effect housed in the double X sex chromosomes could contribute to why the prevalence of hypertension is higher in these populations.

Caeiro, X.E. et al. [164] recently reported that C57BL/6 mice with the XY sex chromosomal complement show a lesser decrease in heart rate induced by Ang II when compared to the response in the XX sex chromosome complement. They also showed involvement of the sex chromosome complement in the baroreflex regulation of heart rate and that the sex chromosome effect contributes to the sex differences in the Ang II-bradycardic baroreflex response. These findings support our previous findings showing a sex chromosome effect on the magnitude of hypertension [39] and other studies suggesting certain brain functions are regulated by sex chromosome independently of the gonadal state [163].

Studies implicate a role for *Sry *in the SHR genetic model of hypertension. Switching the Y chromosome between SHR and the normotensive Wistar-Kyoto (WKY) rat increases arterial pressure by 15-30 mm Hg in WKY and reduces the arterial pressure to a similar degree in SHR [165,166]. Furthermore, the SHR Y chromosome is associated with increased sympathetic nervous system activity. When the *Sry1 *gene was delivered to the WKY kidney, the renal sympathetic nervous system [167] was activated and arterial pressure increased in this normotensive strain. Thus, these studies suggest it is the *Sry *gene on the Y chromosome that is contributing to the hypertension in the SHR. These *Sry *findings in the SHR do not, however, explain the observation of a sex chromosome effect in the four core genotype mice since the magnitude of the hypertension was higher in the gonadectomized XX mice compared to the XY mice independently of whether they were born with testes (+*Sry*) or ovaries (-*Sry*) [39].

While many studies have investigated the role of gonadal hormones on physiological parameters that are known to modulate arterial pressure (e.g., endothelial function, renal function, and the sympathetic nervous system), it is imperative to realize that demonstrating hormonal regulation of one of these parameters in either sex does not mean this mechanism is a major contributor to male-female differences in blood pressure control. Thousands of genes [168] are differentially expressed in male and female tissues and not surprisingly, whole signaling networks are also differentially expressed [169]. A microarray analysis of 23,574 transcripts showed that thousands of genes are differentially expressed between male and female mice in a tissue-specific manner including in the brain, liver, adipose and muscle tissue and that sex differences exist in the quantitative trait loci that control subsets of these genes [168]. While most of this differential gene expression can be attributed to sex differences in the adult gonadal hormone milieu, some differences were due to the sex chromosomes themselves [169]. Thus, comparisons of hormone effects in male and female animals are difficult to interpret given the magnitude of gene expression differences between the sexes. Furthermore, it behooves us to remember that blood pressure is a complex physiological trait that is controlled by whole networks of systems with no one gene acting in a vacuum. It is simply not valid to conclude the hormones are acting via the same mechanism in males and females just because a similar effect on blood pressure is observed in both sexes. Moreover, the underlying pathology, dose or combination of agents, age, and other factors are likely to determine whether the effects of gonadal steroids are beneficial or adverse.

## Conclusions

Sex differences in blood pressure and the prevalence of hypertension are highly conserved across race, ethnicity and country of origin. This robust finding in humans is also observed among animal species including mammals and avian species. While differences in the gonadal steroid profile contribute to the sexual dimorphism in blood pressure control, the sex chromosomes independently of the gonadal hormone milieu also contribute. Gaps in our knowledge regarding sex differences in primary hypertension include the role of T, FSH and LH and the changes in the ratios of these steroids in impacting blood pressure in both men and women across the life span.

Studies comparing male and female differences in a physiological parameter like blood pressure have numerous confounds whereas studies conducted using the four core genotype mouse model are uniquely able to isolate sex chromosome from gonadal hormone effects that occur *in utero *or during development. Alternatively, focusing on gonadal hormone regulation within one sex is a valuable strategy for gaining insights relevant to that specific sex. Moreover, focusing on genes on the Y chromosome that are not found on the X and the role of genes that escape X-inactivation or studying the influence of parental imprinting and mosacisim on arterial pressure is likely to uncover new insights into blood pressure control that may ultimately be used to develop novel targets for the treatment of hypertension and associated disease as well as provide the rationale for treating men and women with antihypertensive regimens that reach maximal optimization in both sexes.

## Competing interests

The authors declare that they have no competing interests.

## Authors' contributions

KS and HJ each contributed to the literature review and the writing of this manuscript. All authors read and approved the final manuscript.

## Authors' information

KS is the director of Center for the Study of Sex Differences in health, aging and disease, and the vice-chair for Research in Department of Medicine at Georgetown University Medical Center.
